# Small cell transformation during acquired resistance to zongertinib in HER2-mutant non-small cell lung cancer: a case report

**DOI:** 10.3389/fonc.2026.1934878

**Published:** 2026-09-25

**Authors:** Danru Zheng, Haiyan Xu, Yan Wang

**Affiliations:** 1 Department of Medical Oncology, National Cancer Center/National Clinical Research Center for Cancer/Cancer Hospital, Chinese Academy of Medical Sciences and Peking Union Medical College, Beijing, China; 2 Department of Comprehensive Oncology, National Cancer Center/National Clinical Research Center for Cancer/Cancer Hospital, Chinese Academy of Medical Sciences and Peking Union Medical College, Beijing, China

**Keywords:** case report, HER2 mutation, non-small cell lung cancer, small cell transformation, zongertinib

## Abstract

**Background:**

Small cell transformation (SCT) is a known acquired resistance mechanism to targeted therapies in non-small cell lung cancer (NSCLC) but has not been reported following HER2 tyrosine kinase inhibitor (TKI) therapy. We report the first case of SCT detected during acquired resistance to zongertinib, a novel selective HER2 TKI, in a patient with HER2-mutant pulmonary enteric adenocarcinoma.

**Case presentation:**

A 55-year-old never-smoking male with advanced pulmonary enteric adenocarcinoma harboring HER2 exon 20 YVMA insertion (p.Y772_A775dup) and concurrent TP53/RB1 co-mutations received multiple prior lines of therapy. He obtained partial response to zongertinib. After six months, neuron-specific enolase (NSE) and pro-gastrin-releasing peptide (ProGRP) rose with disease progression. Rebiopsy of a progressing lesion revealed SCT with 80% SCLC and 20% adenocarcinoma; genomic profiling retained the original HER2 insertion and TP53/RB1 mutations. Following transformation, etoposide plus carboplatin decreased NSE and ProGRP but paradoxically increased carcinoembryonic antigen (CEA). Adding zongertinib reduced CEA, whereas the SCLC component continued to progress. The patient survived 5.6 months after SCT diagnosis.

**Conclusion:**

This is the first report of SCT emerging during acquired resistance to a HER2 TKI. Baseline TP53/RB1 co-mutations may identify patients at higher risk for histologic transformation. Dynamic monitoring of neuroendocrine markers facilitates early detection, and mixed histology warrants individualized strategies targeting both tumor subclones.

## Introduction

Lung cancer remains one of the leading causes of cancer-related mortality worldwide (1). Recent population-based studies in China have highlighted the increasing importance of molecular characterization and precision oncology in lung cancer management (2).

Small cell transformation (SCT) is an established resistance mechanism to targeted therapy in oncogene-driven non-small cell lung cancer (NSCLC). Previous studies show 3–15% of EGFR-mutant NSCLC patients develop SCT after EGFR tyrosine kinase inhibitors (TKIs), with similar cases reported in ALK, ROS1, RET, and BRAF-mutant patients (3).However, no definitive case of SCT following HER2 TKI therapy has been reported to date.

HER2 mutations, particularly exon 20 insertions, represent an emerging actionable molecular subtype of NSCLC. With the recent development of HER2-directed therapies, including HER2 TKIs, understanding mechanisms of acquired resistance in HER2-mutant NSCLC has become increasingly important.

We report a patient with advanced pulmonary enteric adenocarcinoma harboring HER2 exon 20 insertion and TP53/RB1 mutations who was diagnosed with SCT during acquired resistance to zongertinib after multiple lines of therapy, aiming to expand the known resistance spectrum of HER2-targeted therapy and inform clinical practice.

## Case presentation

A 55-year-old never-smoking male was diagnosed with locally advanced left pulmonary enteric adenocarcinoma in September 2018. He received two cycles of pemetrexed plus cisplatin followed by surgery, with a postoperative stage of ypT2aN2M0 (IIIA). Genetic testing revealed HER2 exon 20 YVMA insertion (p.Y772_A775dup, variant allele frequency [VAF] 53.80%), RB1 (p.S347Ffs6, VAF 51.70%), and TP53 (p.Y163C, VAF 65.50%) mutations.

One month after surgery, cervical and mediastinal lymph node metastases developed. He received first-line docetaxel, cisplatin, and endostatin with concurrent radiotherapy. In July 2019, right lung metastases appeared, followed by second-line afatinib plus bevacizumab, with liver metastases occurring 3.4 months later.

Subsequent treatments at our hospital included pyrotinib plus apatinib (progression-free survival [PFS] 6.1 months), poziotinib (PFS 1.3 months), nab-paclitaxel plus bevacizumab (PFS 10.7 months), and mobocertinib plus anlotinib (PFS 12.5 months). Later therapies (nab-paclitaxel, disitamab vedotin, PD-1 inhibitor, S-1, mobocertinib) all progressed within 1–3 months. Plasma next-generation sequencing (NGS) revealed persistent HER2, RB1, and TP53 mutations, with newly acquired CREBBP mutation after fourth-line therapy and PTEN loss after ninth-line therapy (**Figure 1**; **Supplementary Tables 1**, **2**).

**Figure 1:**
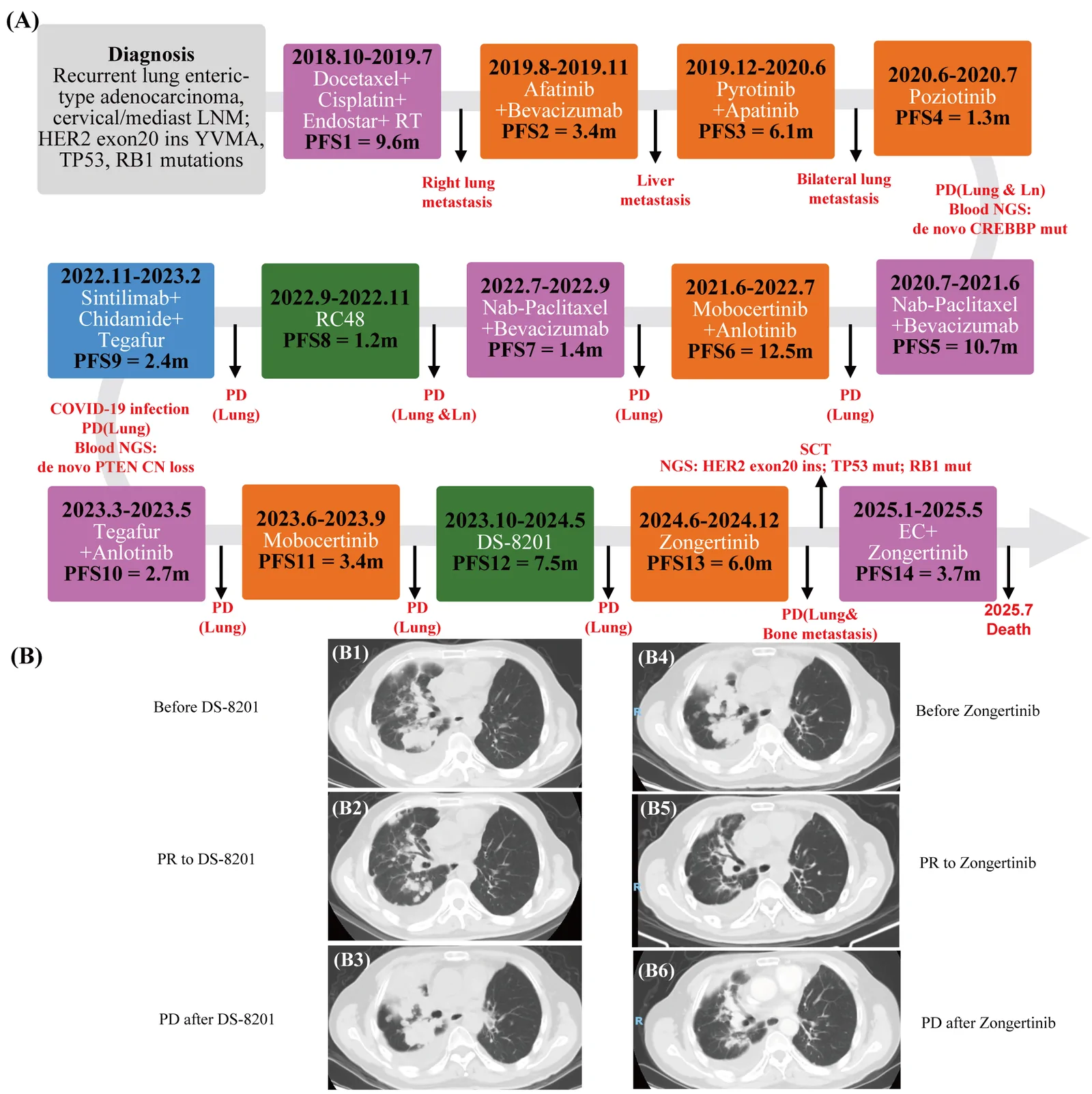
Treatment timeline after disease recurrence and radiological responses. **(A)** Treatment timeline after tumor recurrence. The patient developed recurrent pulmonary enteric-type adenocarcinoma harboring HER2 exon 20 YVMA insertion and concurrent TP53/RB1 mutations in October 2018. Fourteen lines of systemic therapy were given from October 2018 to July 2025. Acquired CREBBP mutation and PTEN loss were detected during treatment, and small cell transformation (SCT) developed was identified during acquired resistance to zongertinib in January 2025. **(B)** Representative axial chest CT images showing tumor radiological changes during DS-8201 and zongertinib. (B1) Baseline imaging DS-8201; (B2) partial response (PR) to DS-8201; (B3) progressive disease (PD) after DS-8201; (B4) baseline imaging before zongertinib; (B5) PR to zongertinib; (B6) PD after zongertinib. HER2, human epidermal growth factor receptor 2; ins, insertion; PFS, progression-free survival; PD, progressive disease; LN, lymph node; NGS, next-generation sequencing; CREBBP, CREB-binding protein; mut, mutation; RC48, disitamab vedotin; PTEN, phosphatase and tensin homolog; CN loss, copy number loss; DS-8201, trastuzumab deruxtecan; SCT, small cell transformation; EC, etoposide plus carboplatin.

In October 2023, he received twelfth-line trastuzumab deruxtecan (T-DXd; DS-8201) and achieved a partial response (PR). However, disease progressed in June 2024. Thirteenth-line zongertinib again resulted in PR, which lasted 6 months until progression with new bone metastases and elevated NSE (41.9 ng/mL) and ProGRP (184.0 pg/mL).

By January 2025, NSE further increased to 103.0 ng/mL and ProGRP to 311.0 pg/mL. Rebiopsy of the lung metastasis showed poorly differentiated carcinoma with 80% small cell carcinoma (Syn, CgA, CD56 partially positive; RB1 protein expression lost; Ki-67 90%+) and 20% adenocarcinoma (Ki-67 70%+) (**Figure 2**; **Supplementary Figure 1, 2**). NGS retained the original HER2 (VAF 56.39%), RB1 (53.79%), and TP53 (66.96%) mutations, with a new BRAF R389C mutation (VAF 8.37%). A diagnosis of SCT was confirmed.

**Figure 2:**
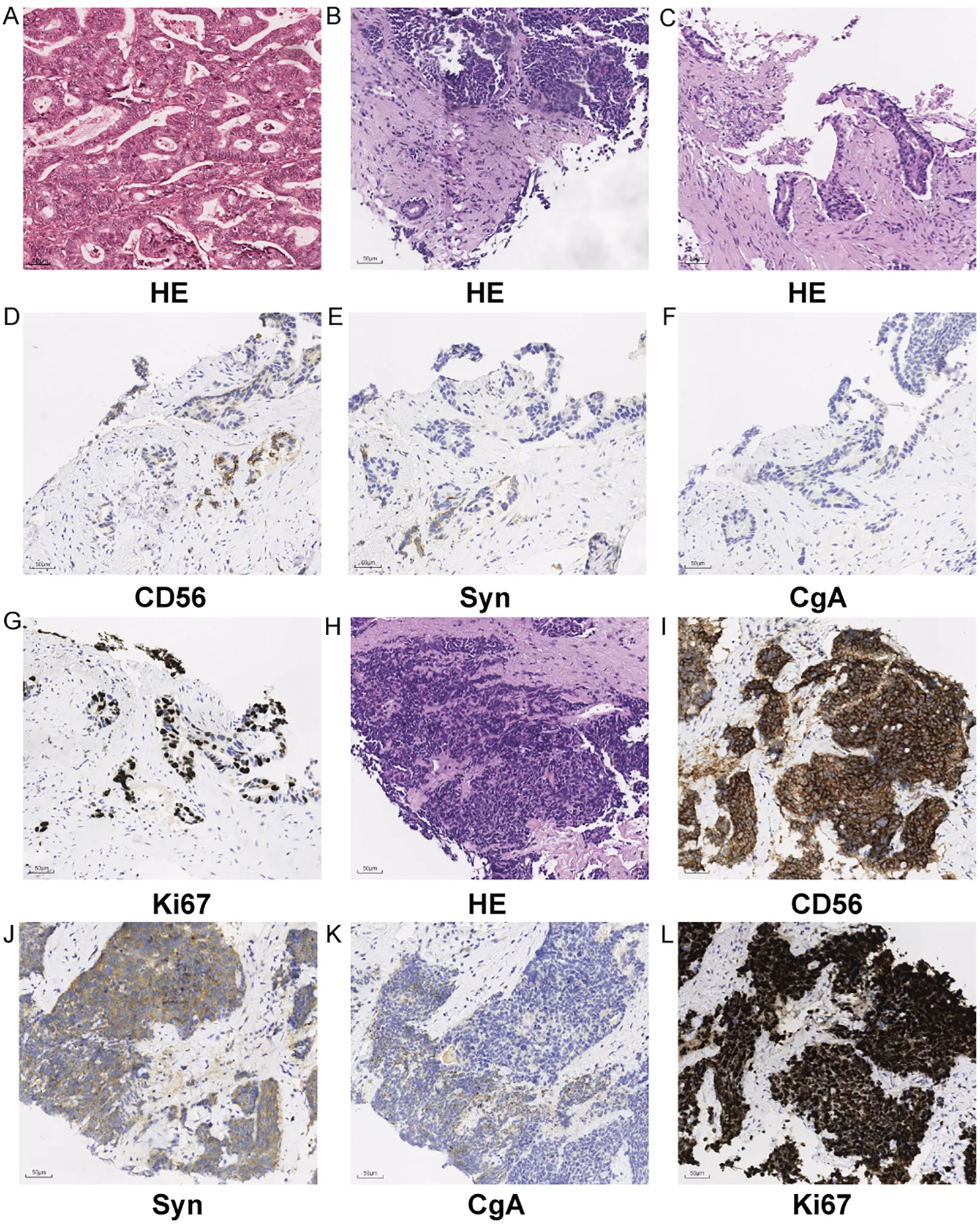
Pathological evaluation of pre- and post-transformation specimens. **(A)** Hematoxylin and eosin (H&E) staining of the primary lung enteric-type adenocarcinoma specimen obtained after surgery. **(B)** H&E staining of the rebiopsy specimen after progression on zongertinib, showing a poorly differentiated carcinoma with mixed histology: approximately 80% small cell carcinoma and 20% adenocarcinoma. **(C–G)** Adenocarcinoma component of the rebiopsy specimen: **(C)** H&E, **(D)** CD56, **(E)** synaptophysin (Syn), **(F)** chromogranin A (CgA), **(G)** Ki-67. **(H–L)** Small cell carcinoma component of the rebiopsy: **(H)** H&E, **(I)** CD56, **(J)** Syn, **(K)** CgA, **(L)** Ki-67. CgA, chromogranin A; H&E, hematoxylin and eosin; SCLC, small cell lung cancer; Syn, synaptophysin.

He then received four cycles of etoposide plus carboplatin (EC). After two cycles, NSE/ProGRP decreased, but CEA progressively increased; subsequent addition of zongertinib reduced CEA but was followed by renewed NSE/ProGRP elevation and radiologic progression (**Figure 3**). Treatment was complicated by grade 4 neutropenia and pulmonary infection. In July 2025, the patient died, with an overall survival of 5.6 months from SCT diagnosis.

**Figure 3:**
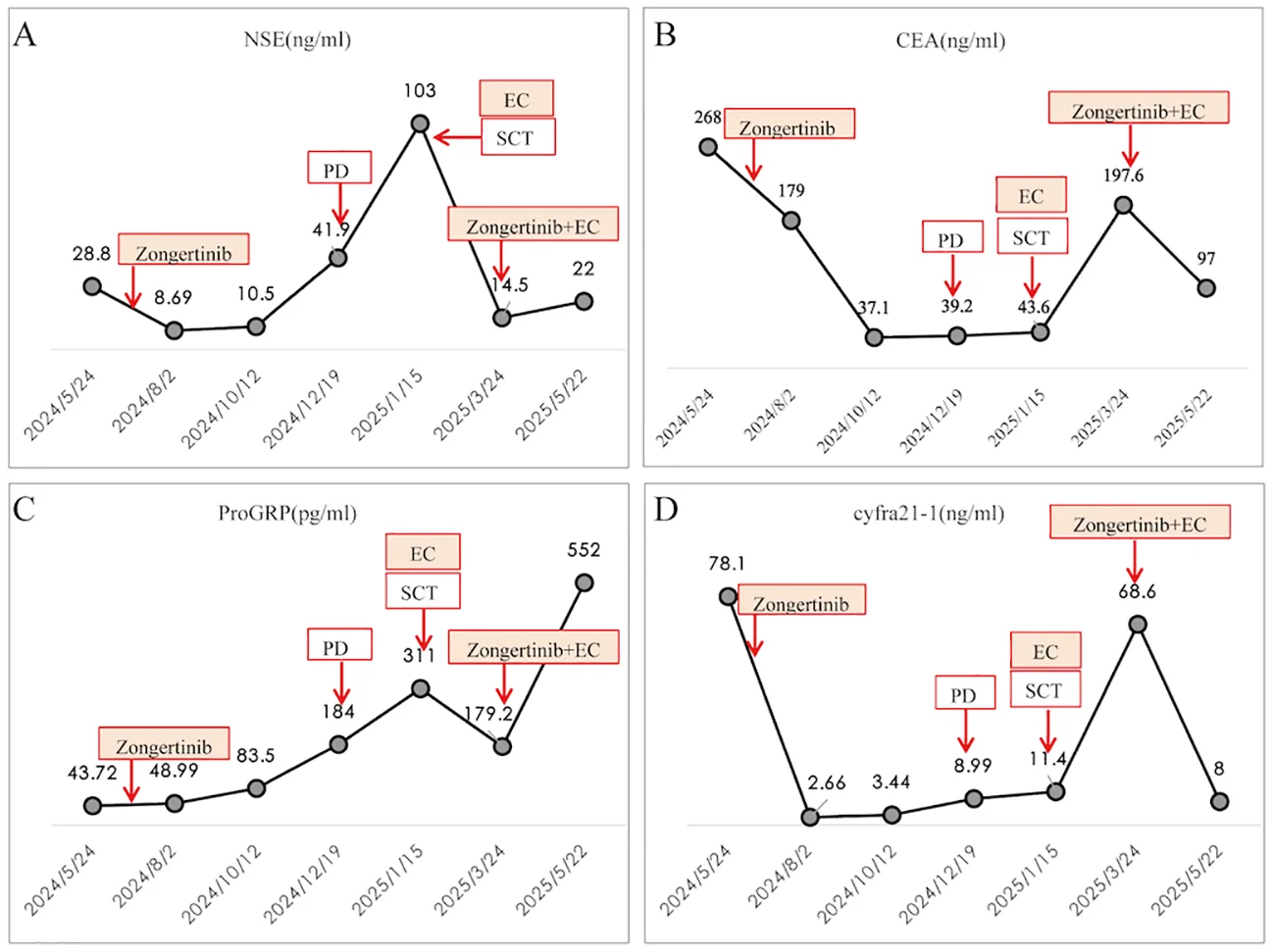
Serial changes in serum tumor markers during zongertinib therapy and after histologically confirmed SCT. During zongertinib monotherapy, NSE and ProGRP levels gradually increased. SCT was confirmed by rebiopsy on January 15, 2025. After switching to etoposide plus carboplatin (EC) chemotherapy, NSE and ProGRP declined while CEA rose markedly. Subsequent addition of zongertinib reduced CEA, followed by renewed elevation of NSE and ProGRP. **(A)** NSE (reference range: <16.3 ng/mL); **(B)** CEA (reference range: <5 ng/mL); **(C)** ProGRP (reference range: <65.7 pg/mL); **(D)** CYFRA21-1 (reference range: <3.3 ng/mL). CEA, carcinoembryonic antigen; EC, etoposide plus carboplatin; NSE, neuron-specific enolase; ProGRP, progastrin-releasing peptide; SCT, small cell transformation.

## Discussion

To our knowledge, this is the first reported case of SCT detected during acquired resistance to a HER2 TKI in NSCLC.

Two main hypotheses explain SCT: tumor heterogeneity and lineage plasticity (3). NSCLC and SCLC have traditionally been considered to arise from distinct epithelial lineages; however, emerging evidence indicates that cellular identity is not fixed and can be reshaped by genetic alterations and therapeutic pressure, enabling non-neuroendocrine tumor cells to acquire neuroendocrine features. NSCLC-to-SCLC transformation is increasingly recognized as an evolutionary process driven by lineage plasticity, involving coordinated genetic, epigenetic, and transcriptional reprogramming rather than a single molecular event (4, 5). In this case, although the presence of mixed histology in a single rebiopsy cannot completely exclude pre-existing tumor heterogeneity, several findings suggest that lineage plasticity may have contributed to SCT development. First, the patient achieved a partial response lasting 6 months to zongertinib, arguing against a clinically significant pre-existing SCLC clone at treatment initiation. A dominant SCLC component would be less likely to derive substantial benefit from HER2 inhibition and could have resulted in earlier progression. Second, the transformed tumor retained the original HER2 mutation and TP53/RB1 alterations, without additional commonly reported SCLC-associated alterations (e.g., MYC, MYCL, or SOX2 amplifications), consistent with SCT reported in EGFR-mutant NSCLC (4, 6); Third, neuroendocrine markers (NSE and ProGRP) remained normal before zongertinib treatment but increased during acquired resistance, suggesting the emergence of neuroendocrine differentiation under therapeutic pressure. Nevertheless, given the extensive prior treatment exposure, we cannot exclude the contribution of cumulative selective pressures from earlier therapies. Therefore, SCT in this case should be considered as an evolutionary event emerging during acquired resistance to zongertinib, potentially facilitated by sustained HER2-targeted selective pressure, rather than a transformation solely attributable to zongertinib.

Longitudinal genomic analysis provided additional insights into the evolutionary trajectory of this tumor. The persistent presence of HER2 exon 20 insertion and TP53/RB1 alterations across multiple disease stages supports the clonal relationship between the original HER2-mutant adenocarcinoma and the transformed SCLC component. During treatment, additional alterations, including CREBBP mutation and PTEN loss, emerged, and further genomic alterations, including BRAF R389C mutation, were detected at the time of SCT diagnosis, reflecting continued clonal evolution under therapeutic pressure. These findings suggest that SCT in this case likely resulted from a stepwise evolutionary process involving both baseline genomic vulnerability and subsequent acquired alterations.

Baseline TP53/RB1 mutations are known to increase SCT risk. Offin et al. reported a six-fold higher risk of SCT in EGFR/TP53/RB1 triple-mutant NSCLC (7). Consistently, our case suggests HER2-mutant NSCLC with concurrent TP53/RB1 mutations may represent a subgroup with increased susceptibility to histologic transformation. However, RB1/TP53 inactivation alone may be insufficient; additional cooperating events are needed. Beyond baseline alterations, sequential acquisition of CREBBP mutation and PTEN loss during treatment may have further contributed to transformation susceptibility, consistent with prior reports implicating CREBBP, PTEN, PIK3CA, and MYC as potential co-drivers (8). CREBBP alterations may influence epigenetic regulation, whereas PTEN loss can activate the PI3K/AKT/mTOR pathway, which has been implicated in promoting neuroendocrine differentiation and lineage plasticity during SCT (4).

Therapeutically, SCT remains a major clinical challenge, and no established standard treatment strategy currently exists. Most transformed tumors are treated according to SCLC treatment principles, with platinum–etoposide chemotherapy being the most commonly used regimen; however, responses are generally transient. Given that transformed tumors frequently retain the original oncogenic driver alterations, combination strategies integrating chemotherapy with continued targeted inhibition have been explored. Clinical trials investigating the combination of etoposide plus platinum with EGFR-TKIs to delay or prevent transformation in EGFR/TP53/RB1 triple-mutant patients are ongoing (e.g., NCT03567642). Whether similar strategies apply to HER2-mutant high-risk patients remains unknown and requires further investigation.

Tumor markers play a valuable role in predicting histological transformation. In our case, NSE and ProGRP remained within the normal range before zongertinib treatment but increased during acquired resistance, suggesting emerging neuroendocrine differentiation. Therefore, repeat biopsy should be considered when neuroendocrine markers rise and disease progresses. After SCT diagnosis, CEA levels rose during EC chemotherapy but declined upon reintroduction of zongertinib, suggesting residual adenocarcinoma dependence on HER2 signaling. Whether to continue TKI after SCT remains controversial (3, 9). Our case suggests that in patients with mixed histology tumors, continuing TKI therapy may help delay overall disease progression. Additionally, over 90% of SCT samples express DLL3, making DLL3-targeted agents (e.g., tarlatamab) potentially promising for transformed SCLC (9). Other emerging therapeutic approaches targeting SCT-associated vulnerabilities, including epigenetic regulators (e.g., EZH2 and HDAC inhibitors), lineage plasticity-related mechanisms (e.g., NOTCH signaling), and other molecular dependencies of transformed tumors, are currently under investigation and may provide additional therapeutic opportunities for SCT (4, 5).

Another notable clinical finding is the potential benefit of sequential therapy with a HER2 ADC followed by HER2 TKIs. After failing multiple lines of chemotherapy and pan-HER2 TKIs, the patient still derived meaningful benefit from T-DXd, achieving a PR with a PFS of 7.5 months. Moreover, upon progression on T-DXd, the patient subsequently responded to zongertinib, with a further PFS of 6 months. This observation suggests that the resistance mechanisms to HER2 ADCs and HER2 TKIs are not completely overlapping. Preclinical data have demonstrated that HER2 ADC-resistant cells maintain comparable HER2 expression and intact HER2 signaling, and remain sensitive to multiple HER2 TKIs (10, 11). Collectively, these data imply that tumors progressing on T-DXd remain largely HER2-signaling dependent, thereby providing a clinical rationale for sequential use of HER2 TKIs after failure of HER2 ADCs.

Our study has limitations, including single-center case report, single-lesion rebiopsy, and multiple prior therapies precluding exclusive attribution of transformation to zongertinib. Nonetheless, this first report of SCT emerging during HER2 TKI treatment expands the resistance spectrum of HER2-targeted therapy and highlights the need for vigilant monitoring and individualized treatment.

## Conclusion

This case represents the first reported SCT detected during HER2 TKI treatment in HER2-mutant NSCLC. Dynamic monitoring of NSE/ProGRP facilitates early detection of transformation. Timely repeat biopsy is crucial for suspected cases. Post-transformation management should consider targeting both SCLC and residual HER2-dependent adenocarcinoma.

## Data Availability

The original contributions presented in the study are included in the article/**Supplementary Material**. Further inquiries can be directed to the corresponding author.

## References

[1] Leiter A Veluswamy RR Wisnivesky JP . The global burden of lung cancer: current status and future trends. Nat Rev Clin Oncol. (2023) 20:624–39. doi: 10.1038/s41571-023-00798-3 37479810

[2] Xu K Wang H Li S Zhao L Liu X Liu Y . Changing profile of lung cancer clinical characteristics in China: Over 8-year population-based study. Chin Med J Pulm Crit Care Med. (2023) 1:188–94. doi: 10.1016/j.pccm.2023.08.006 39171125 PMC11332861

[3] Giaccone G He Y . Current knowledge of small cell lung cancer transformation from non-small cell lung cancer. Semin Cancer Biol. (2023) 94:1–10. doi: 10.1016/j.semcancer.2023.05.006 37244438

[4] Dai X Hao Y Hong J Yuan J Chen C Huo Z . Molecular mechanisms and clinical insights in transformed small cell lung cancer: a narrative review. Trans Lung Cancer Res. (2025) 14:3233–48. doi: 10.21037/tlcr-2025-165 40948831 PMC12432677

[5] Mukherjee A Mukherjee G Das S . Transformation of NSCLC to SCLC: Insights into molecular alterations, tumor origin, and therapeutic advancements. Onco. (2026) 6:36. doi: 10.3390/onco603003630654563

[6] Gu L Zhang B Zhang D Jian H . Clinical and genetic characteristics of EGFR-mutant lung adenocarcinoma transformed SCLC. J Clin Oncol. (2021) 39:e20588–8. doi: 10.1200/JCO.2021.39.15_suppl.e2058842148471

[7] Offin M Chan JM Tenet M Rizvi HA Shen R Riely GJ . Concurrent RB1 and TP53 alterations define a subset of EGFR-mutant lung cancers at risk for histologic transformation and inferior clinical outcomes. J Thorac Oncol. (2019) 14:1784–93. doi: 10.1016/j.jtho.2019.06.002 31228622 PMC6764905

[8] Sivakumar S Moore JA Montesion M Sharaf R Lin DI Colón CI . Integrative analysis of a large real-world cohort of small cell lung cancer identifies distinct genetic subtypes and insights into histologic transformation. Cancer Discov. (2023) 13:1572–91. doi: 10.1158/2159-8290.Cd-22-0620 37062002 PMC10326603

[9] Saalfeld FC Möller J Christopoulos P Wenzel C Rasokat A Wang XA . Small cell transformation in EGFR-mutated non-small cell lung cancer: DLL3 expression and efficacy of immune checkpoint inhibitors or tyrosine kinase inhibitors combined with chemotherapy. Eur J Cancer. (2024) 213:115065. doi: 10.1016/j.ejca.2024.115065 39423775

[10] Nilsson MB Le X Poteete A Yu X He J Huang Q . Loss of payload sensitivity and other mechanisms of resistance to T-DXd in HER2-mutant NSCLC: Implications for subsequent responsiveness to HER2 TKIs. J Thorac Oncol. (2026) 21:103555. doi: 10.1016/j.jtho.2026.01.007 41548807 PMC13169646

[11] Murase Y Nanjo S Arai S Kondo S Koba H Liu Y . Therapeutic strategies to overcome payload resistance of trastuzumab deruxtecan in HER2-positive cancers. Cancer Sci. (2026) 117:996–1009. doi: 10.1111/cas.70319 41548044 PMC13045244

