# Supplementary material for: Small cell transformation during acquired resistance to zongertinib in HER2-mutant non-small cell lung cancer: a case report
Source: Front Oncol. 2026 Sep 25;16:1934878. doi: 10.3389/fonc.2026.1934878 (PMC13649505; doi:10.3389/fonc.2026.1934878)
Supplement: Supplementary file 1 [file DataSheet1.docx]

**Supplementary Table 1. Detailed treatment history and clinical outcomes during the disease course**

| **Line** | Treatment regimen | Treatment period | **Dose** | Best response | PFS | Toxicities | Reason for discontinuation |
| --- | --- | --- | --- | --- | --- | --- | --- |
| 1st-line | Docetaxel + cisplatin + endostar with concurrent radiotherapy | 2018/10/12–2019/7/29 | NR | PR | 9.6 months | NR | Right lung metastases |
| 2nd-line | Afatinib+ bevacizumab | 2019/8/17–2019/11/27 | Afatinib 30mg qd+bevacizumab 500mg q3w | SD | 3.4 months | Diarrhea、rash、grade 1 increased creatinine | liver metastases |
| 3rd-line | Pyrotinib + apatinib | 2019/12/9–2020/6/11 | Pyrotinib 320mg qd + apatinib 250mg qd | PR | 6.1 months | Diarrhea、hypertension、decreased appetite、grade 2 increased creatinine | Progression in bilateral lungs |
| 4th-line | Poziotinib | 2020/6/19–2020/7/31 | NR | PD | 1.3 month | NR | Progression in bilateral lungs |
| 5th-line | Nab-paclitaxel + bevacizumab，bevacizumab maintenance | 2020/7/31–2021/6/21 | NR | PR | 10.7 months | NR | Progression in bilateral lungs |
| 6th-line | Mobocertinib + anlotinib | 2021/6/23–2022/7/7 | Mobocertinib 160mg qd+ anlotinib 8 mg d1-14/q3w | PR | 12.5 months | Diarrhea、renal dysfunction、grade 1 increased creatinine | Progression in bilateral lungs |
| 7th-line | Nab-paclitaxel + bevacizumab | 2022/7/20–2022/9/1 | Nab-paclitaxel 200mg d1,d8 + bevacizumab 500 mg d1 q3w | PD | 1.4 month | Febrile neutropenia、grade 4 neutropenia、grade 1 increased aspartate aminotransferase | Progressive pulmonary and abdominal lymph node metastases |
| 8th-line | Disitamab vedotin (RC48) | 2022/9/28–2022/11/3 | NR | PD | 1.2 month | Grade 1 increased aspartate aminotransferase、grade 1 increased creatinine | Progression in bilateral lungs |
| 9th-line | Sintilimab + chidamide + tegafur | 2022/11/18–2023/1/31 | Sintilimab 200mg d1/q21d+ chidamide 20mg biw+ tegafur 60mg d1-14/q3w | PD | 2.4 month | COVID-19 infection, pneumonia | Treatment interruption and subsequent progression |
| 10th-line | tegafur + anlotinib | 2023/3/1–2023/5/22 | tegafur 40 mg bid+ anlotinib 8 mg d1-14/q3w | SD | 2.7 months | Ischemic stroke | Progression in bilateral lungs |
| 11th-line | Mobocertinib | 2023/6/6–2023/9/18 | NR | SD | 3.4 months | Diarrhea, rash, oral ulceration | Progression in bilateral lungs |
| 12th-line | Trastuzumab deruxtecan (DS-8201) | 2023/10/9–2024/5/24 | DS-8201 200mg q3w | PR | 7.5 months | Fatigue、constipation | Progression in bilateral lungs |
| 13th-line | Zongertinib | 2024/6/21–2024/12/21 | Zongertinib 180 mg qd | PR | 6.0 months | NR | Progression in bilateral lungs，bone metastases |
| 14th-line | Etoposide + carboplatin | 2025/1/31–2025/3/30 | Etoposide 430mg q3w+ carboplatin 380mg q3w | SD(neuroendocrine markers decreased, adenocarcinoma markers increased) | 3.7 months | Grade 4 neutropenia; bacterial infection | Progression in right lung |
|  | Etoposide + carboplatin+Zongertinib | 2025/4/1–2025/5/24 | NR | SD(CEA decreased, NSE/ProGRP increased) |  |  |  |

**Abbreviations:** NR: Not reported; PR: Partial response; PFS: Progression-free survival; SD: Stable disease; PD: Progressive disease; qd: once daily; q3w: every 3 weeks; q21d: every 21 days; biw: twice weekly; bid: twice daily; d1: day 1; d8: day 8; d1–14/q3w: days 1 to 14 every 3 weeks; Grade: CTCAE grade; COVID-19: Coronavirus Disease 2019; CEA: Carcinoembryonic antigen; NSE: Neuron-specific enolase; ProGRP: Pro-gastrin-releasing peptide; RC48: Disitamab vedotin; DS-8201: Trastuzumab deruxtecan

Supplementary Table 2. Longitudinal genomic alterations during disease evolution and treatment

| **Date** | **Sample type** | **Gene** | **Variant type** | **Protein change** | **VAF/CN** |
| --- | --- | --- | --- | --- | --- |
| 2018/9/1 | Surgical specimen | ERBB2 | In-frame insertion | p.Y772_A775dup (exon 20 YVMA insertion) | VAF 53.80% |
|  |  | RB1 | Frameshift mutation | p.S347Ffs*6 | VAF 51.70% |
|  |  | TP53 | Missense mutation | p.Y163C | VAF 65.50% |
| 2020/7/1 | Plasma ctDNA | ERBB2 | In-frame insertion | p.Y772_A775dup | VAF 8.60% |
|  |  | RB1 | Frameshift mutation | p.S347Ffs*6 | VAF 5.10% |
|  |  | TP53 | Missense mutation | p.Y163C | VAF 10.70% |
|  |  | CREBBP | Missense mutation | p.S1067N | VAF 1.10% |
| 2022/11/1 | Plasma ctDNA | ERBB2 | In-frame insertion | p.Y772_A775dup | VAF 1.45% |
|  |  | RB1 | Frameshift mutation | p.S347Ffs*6 | VAF 0.70% |
|  |  | TP53 | Missense mutation | p.Y163C | VAF 1.11% |
| 2023/2/1 | Plasma ctDNA | ERBB2 | In-frame insertion | p.Y772_A775dup | VAF 72.72% |
|  |  | RB1 | Frameshift mutation | p.S347Ffs*6 | VAF 72.25% |
|  |  | TP53 | Missense mutation | p.Y163C | VAF 79.24% |
|  |  | PTEN | Copy number loss | CN loss (CN=1.0) | — |
|  |  | CDH1 | Copy number loss | CN loss (CN=1.0) | — |
| 2025/1/1 | Tumor biopsy | ERBB2 | In-frame insertion | p.Y772_A775dup | VAF 56.39% |
|  |  | RB1 | Frameshift mutation | p.S347Ffs*6 | VAF 53.79% |
|  |  | TP53 | Missense mutation | p.Y163C | VAF 66.96% |
|  |  | BRAF | Missense mutation | p.R389C | VAF 8.37% |
|  |  | CDK8 | Missense mutation | p.T229M | VAF 3.69% |
|  |  | FAT1 | Missense mutation | p.N4485T | VAF 25.44% |
|  |  | WRN | Missense mutation | p.F684L | VAF 14.54% |

**Abbreviations:** CN, copy number; ctDNA, circulating tumor DNA; ERBB2, erb-b2 receptor tyrosine kinase 2; VAF, variant allele frequency; YVMA, Y772_A775dup.

**Supplementary Figure 1. Immunohistochemical characterization of the transformed tumor**

(A–D) Immunohistochemical staining of the rebiopsy specimen after SCT, showing expression of neuroendocrine markers

(A) CD56, (B) synaptophysin (Syn), (C) insulinoma-associated protein 1 (INSM1), and (D) chromogranin A (CgA).

**Supplementary Figure 2. Postoperative pathological and immunohistochemical characterization of the primary tumor specimen.**

Representative hematoxylin and eosin (H&E) staining and immunohistochemical findings of the postoperative primary tumor specimen. (A) H&E staining at ×2 magnification. (B–F) Representative images at ×10 magnification showing H&E staining and immunohistochemical staining for TTF-1, Napsin A, CDX2, and CK20, respectively. (G–L) Representative images at ×20 magnification showing immunohistochemical staining for SATB2, H&E staining, TTF-1, Napsin A, CDX2, and CK20, respectively.

Abbreviations:

H&E, hematoxylin and eosin; TTF-1, thyroid transcription factor-1; Napsin A, napsin A; CDX2, caudal-type homeobox 2; CK20, cytokeratin 20; SATB2, special AT-rich sequence-binding protein 2.
